# A handheld HIV detection platform using paper-based sample preparation and real-time isothermal amplification

**DOI:** 10.1038/s41378-024-00822-1

**Published:** 2024-11-29

**Authors:** George Adedokun, Gurjit Sidhu, Morteza Alipanah, Gary P. Wang, Z. Hugh Fan

**Affiliations:** 1 Interdisciplinary Microsystems Group, Department of Mechanical & Aerospace Engineering, University of Florida, Gainesville, FL 32611 USA; 2 Division of Infectious Diseases and Global Medicine, Department of Medicine, College of Medicine, University of Florida, Gainesville, FL 32610 USA; 3 Medical Service, North Florida/South Georgia Veterans Health System, Gainesville, FL 32608 USA; 4 Purrit Family Department of Biomedical Engineering, University of Florida, Gainesville, FL 32611 USA

**Keywords:** Chemistry, Engineering

## Abstract

Early and accurate diagnosis of human immunodeficiency virus (HIV) infection is essential for timely initiation of antiretroviral therapy (ART) and prevention of new infections. However, conventional nucleic-acid-based tests for HIV detection require sophisticated laboratory equipment and trained personnel, which are often unavailable at the point-of-care (POC) or unaffordable in resource-limited settings. We report our development of a low-cost, integrated platform for POC testing of HIV. The platform integrates viral nucleic acid extraction on a paper substrate and reverse transcription loop-mediated isothermal amplification (RT-LAMP) in a portable, battery-powered heating device with real-time detection. The platform does not require laboratory infrastructure such as power outlets. The assay showed a detection limit of 30 copies/mL of HIV RNA in 140 μL human serum or 4 copies/reaction using 50 μL human serum, with no cross-reactivity with hepatitis C virus (HCV). We validated the platform using both plasma samples spiked with HIV and clinical samples from HIV-positive individuals, and compared it with standard laboratory assays based on polymerase chain reaction (PCR). These results demonstrate the feasibility of our platform for HIV testing at the POC.

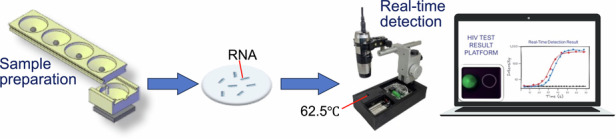

## Introduction

Human immunodeficiency virus (HIV) is one of the most important public health challenges in the world, with significant social and economic impacts. According to the World Health Organization, about 38.4 million people were living with HIV as of 2021, and 650,000 people died from AIDS-related illnesses in that year^1^. HIV is transmitted through sexual contact, blood transfusion, sharing of contaminated needles, and from mother to child during pregnancy, delivery, or breastfeeding^2–4^.

Diagnosing HIV infection is crucial for initiating timely antiretroviral therapy (ART), reducing morbidity and mortality, and preventing further transmission^5^. However, HIV diagnosis is challenging because the symptoms are often nonspecific or absent, especially in the early stages of infection^6–8^. Moreover, many people who are infected with HIV are unaware of their status and may unknowingly transmit the virus to others. Studies have shown that people who are unaware of their HIV status have a higher risk of transmission than those who are aware and on ART^9^. This is partly because the viral load is highest in the acute phase of infection, which occurs within the first few weeks after exposure^10^. Therefore, detecting HIV infection at an early stage, especially those at low-cost and accessible at the point-of-care (POC), can have significant benefits for individual and public health.

The United Nations Joint Programme on HIV/AIDS (UNAIDS) has set a global target of 90-90-90 by 2030; 90% of people living with HIV should know their status, 90% of those diagnosed should receive ART, and 90% of those on ART should achieve viral suppression^11^. The success of this target heavily depends on the availability and accessibility of HIV testing services^12^. Rapid HIV tests that are based on the detection of antibodies against HIV, including OraQuick Advance Rapid HIV-1/2 antibody test®^13^, Alere Determine™ HIV-1/2 Ag/Ab Combo^14^, INSTI™ HIV-1/HIV-2 Rapid Antibody (INSTI) test^15^, Chembio DPP® HIV-1/2 Assay^16^, Reveal G4 Rapid HIV-1 Antibody Test^17^, Uni-Gold Recombigen HIV-1/2^18^, have facilitated self-testing and POC testing in non-clinical and low-resource settings. These tests have several advantages such as simplicity, low cost, portability, and fast results. Self-testing and POC testing have also been shown to increase the uptake of testing, reduce stigma and discrimination, and decrease loss to follow-up^19–21^.

However, antibody-based HIV tests have limitations, such as low sensitivity during acute infection when antibodies are not yet detectable (seroconversion period), cross-reactivity with other infections or conditions, and inability to distinguish between acute and chronic infection^22^. Therefore, nucleic acid testing (NAT) is considered the gold standard for diagnosis of acute HIV infection because it can directly detect the presence of HIV RNA in the sample^23,24^. NAT can also provide quantitative information on viral load, which is useful for monitoring treatment response and drug resistance^25,26^. However, NATs such as Cepheid GeneXpert®^27^, Abbott RealTime, and Cobas® 6800/8800 System^28^ are expensive, complex, time-consuming, and require trained personnel^29^. These factors limit their use in resource-limited settings and POC detection.

There is a need for low-cost, simple, and rapid methods for HIV NAT, especially in resource-limited settings where access to conventional laboratory testing is limited or unavailable. Isothermal amplification methods have emerged as promising alternatives to polymerase chain reaction (PCR) for NAT because they can amplify nucleic acids at a constant temperature without the need for thermal cycling^30^. Commercial products include the NucliSENS EasyQ® (based on nucleic acid sequence-based amplification or NASBA)^31^, Alethia (Meridian Bioscience) (based on loop-mediated isothermal amplification or LAMP)^32^, Quidel Solana (based on helicase-dependent amplification or HDA)^33^, and TwistDx (based on recombinase polymerase amplification or RPA). Among these methods, RT-LAMP (reverse transcription LAMP) has been widely used for the detection of various pathogens including HIV^34,35^. RT-LAMP can amplify RNA targets with high specificity and sensitivity in less than 30 min using a simple heating device and colorimetric indicators^36,37^. LAMP was invented by Notomi et al. and its schematic representation of nucleic acid amplification steps can be found in their pioneering works^38,39^.

Another advantage of RT-LAMP is its lower sensitivity to biological inhibitors^40,41^, allowing for direct amplification from minimally lysed samples and reducing the time and cost of sample preparation. However, direct amplification is not sufficient for high-sensitivity detection of nucleic acids, as the lysis step alone may introduce contaminants that affect the RT-LAMP assay, as shown by Bau and his research group^42^. Therefore, several researchers have developed devices that integrate sample preparation with isothermal amplification for early detection of HIV. For example, Bashir and his colleagues^43^ designed a device that detects HIV from minimally lysed samples, but with a tradeoff in sensitivity. Liu et al. ^44^ also created an HIV NAT-on-USB device that combines sample preparation with RT-LAMP.

Paper-based diagnostic devices are another potential sample preparation solution for POC detection of various diseases including HIV^45–47^. Paper is an attractive material for diagnostic applications because it is cheap, biodegradable, widely available, easy to manipulate, and compatible with various biological samples. Linnes and her colleagues^48^ used a paper device to prepare samples and integrate them with RT-LAMP and lateral flow immunoassay (LFIA). We aim to improve the sensitivity of HIV detection by enriching RNA from extracted samples on a paper pad without elution. This approach differs from other methods that typically involve elution and have been reported in the literature for HIV detection. We have also integrated the sample preparation with colorimetric and real-time detection of HIV at the POC.

We report our development of an integrated platform for HIV detection that uses paper as an inexpensive material for nucleic acid extraction. The viral RNA is enriched on the paper-based device and amplified by RT-LAMP in a battery-operated heater. The amplified RNA is detected by either a colorimetric assay or a real-time imaging setup. We implemented a liquid handling scheme enabled by ball valves in our 3D-printed device, allowing for the storage and sequential delivery of reagents required for sample preparation. Our platform showed robust performance, detecting HIV in human serum at concentrations as low as 30 copies/mL. We verified the utility of our platform using clinical plasma samples from HIV-positive individuals and the results are in agreement with those using conventional laboratory assays. We also demonstrated the specificity of our assay using clinical samples containing hepatitis C virus (HCV). Our platform shows potential for low-cost, accurate, equipment-free HIV testing at the POC.

## Materials and methods

### HIV Samples

HIV RNA samples were prepared by extraction from HIV-1 RF Virus (ARP-2803) obtained from the National Institutes of Health (NIH) HIV Reagent Program. Additionally, HIV-1 particles (ARP-3443), which contain 150,000 copies of HIV-1 RNA/mL, were also acquired from the NIH HIV Reagent Program. The ARP-3443 stock is a collection of HIV subtype B derived from five distinct HIV-infected donors who were treatment-naive. 1.25 mL of ARP-3443 was spiked into HIV-negative human serum; the resultant solution was stored at −80 °C after aliquoting. This stock solution was serially diluted in phosphate-buffered saline (PBS) and the resulting samples with appropriate HIV copy numbers were used for experiments.

### RT-LAMP Reaction

The sequences of primers were obtained from the literature^49^ and are listed in Table S1 in the *Supplementary Information*. The HIV RT-LAMP 10× primer mix contained 8 μM each of forward inner primer (FIP) and backward inner primer (BIP), 1 μM each of forward external primer (F3) and backward external primer (B3), and 2 μM each of forward loop primer (FLP) and backward loop primer (BLP), except in those experiments for studying the effects of primer concentrations.

The RT-LAMP mix of 25 μL contained 1.4 mM deoxy-ribonucleoside triphosphates (dNTPs), 2.5 μL of 10× isothermal amplification buffer, 2.5 μL of 10× concentrated primer mix, 6 mM of MgSO_4_, 8 U of a Bst 2.0 WarmStart DNA polymerase from New England Biolabs (NEB, Ipswich, MA, USA), 7.5 U of a WarmStart RTx reverse transcriptase (NEB), 0.5 units of the antarctic thermolabile uracil-DNA glycosylase (UDG), 0.7 mM deoxyuridine triphosphate (dUTP), and nuclease-free water (not DEPC-treated) (ThermoFisher, MA, USA). Adding UDG and dUTP to the reaction mix eliminates the possibility of carryover contamination, reducing false positives and non-specific amplification^50,51^.

Negative controls were included in every experiment unless otherwise stated. The no template control (NTC) comprised 25 μL of RT-LAMP mix without any RNA, virus particles, or HIV-positive samples.

To carry out real-time amplification of RT-LAMP for determining appropriate reaction time, we added a mixture of 25 µL of RT-LAMP reagent and HIV RNA to a 96-well microplate and subjected them to amplification at 62.5 °C for 60 min using an Applied Biosystems QuantStudio 3 Real-Time PCR System (ThermoFisher Scientific). Concurrently, an NTC was introduced to each test as a control. Either 4 µM of SYTO™ 9 green fluorescent nucleic acid stain (ThermoFisher Scientific) or SYBR Green I nucleic acid gel stain in dimethyl sulfoxide (Thermo Fisher Scientific) was used for fluorescence or colorimetric detection.

### Sample Preparation Device

Sample preparation was carried out using a handheld device previously reported^50^. The key differences of this work from the previous one include the following three aspects. First, the device previously designed for Zika virus detection was adapted for HIV detection in this work. Significant modifications are required since six primers used in RT-LAMP must be specific to HIV. The assay is then tested for the sensitivity and specificity, which are different between ZIKV and HIV because their possible interferent pathogens are different. Secondly, we replaced colorimetric detection with a real-time detector for viral load quantification. The integration of the assay with the real-time detector involves the significant innovation in engineering, including enabling heater/controller/optics to be portable and battery powered. Compared to the colorimetric detection, the real-time method provides quantitative information on viral load, which is crucial for HIV management. Thirdly, the significance of this work is represented by clinical validation of the platform using real-world samples, illustrating its potential impact on global health challenges.

**Fig. 1:**
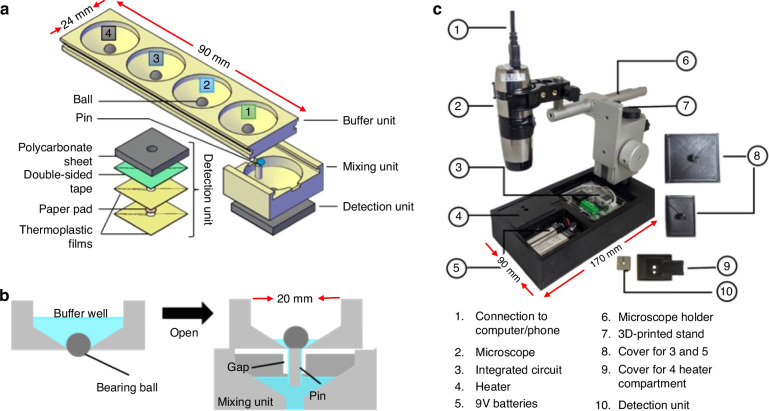
An HIV detection platform combining paper-based sample preparation with real-time isothermal amplification. **a** The handheld device is composed of three main components: a buffer unit, a mixing unit, and a detection unit. The buffer unit contains four reservoirs for housing lysis buffer (1), binding buffer (2), and 2 wash buffers (3) and (4), and the sequential release of these buffers are controlled by ball-based valves. The buffer unit and mixing unit can slide with each other, and the mixing unit is integrated with the detection unit through a protrusion beneath the mixing unit. The detection unit contains a paper pad laminated between two layers of the thermoplastic film as illustrated in the exploded view on the bottom left. **b** Cross-sectional view of the valve. The valve closes with a bearing ball acting as a plug for the reservoir. As the buffer unit slides along the mixing unit. The valve is activated by a pin in the mixing unit, which pushes the ball up to release the liquid from the reservoir when the pin is aligned with the ball. **c** Image of a fully assembled, real-time detector consisting of a handheld microscope mounted on a 3D-printed stand, which houses three compartments at its base for a heater ④, batteries ⑤, and an integrated electrical circuit for controlling temperature ③

The device is 3D-printed containing paper-based components for RNA enrichment/purification. As shown in Fig. 1a and Figure S1 in *Supplementary Information*, the device has three parts: a buffer unit, a mixing unit, and a detection unit. The mixing unit was integrated with the detection unit by inserting the bottom protrusion of the mixing unit into the center hole of the detection unit. The mixing unit was attached to the buffer unit using a sliding track created at the edges of the buffer unit, allowing the mixing unit to be manually slid along the buffer unit.

The buffer unit has 4 wells that hold the lysis buffer, binding buffer, wash buffer 1, and wash buffer 2. Each well consists of a ball-based valve for reagent storage and the sequential delivery of buffers from the buffer unit to the mixing unit. The mixing unit is designed with a pin that pushes up the ball to release the reagents into the mixing unit as it slides (Fig. 1b). The valving mechanism is similar to a ballpoint pen, in which ink is delivered onto paper when the ball at the tip of the pen is pressed while writing.

The detection unit, which is an assembly of a polycarbonate well layer (McMaster-Carr, Elmhurst, IL) and a chromatography paper (Fisher Scientific) laminated between two thermoplastic lamination films (Lamination Plus, Kaysville, UT, USA), is attached to the bottom of the mixing unit with double-sided adhesive tape (3M, R. S. Hughes, Sunnyvale, CA) as shown at the bottom left of Fig. 1a.

### Sample preparation method

The sample preparation protocol used in this work is adapted from the QIAamp Viral RNA mini kit (QIAGEN, Valencia, CA, USA) but without the use of lab equipment such as a centrifuge machine. The cellulose chromatography paper was used to replace the spin column for solid-phase extraction of RNA because it proved effective. Additionally, it demonstrated a lower limit of detection for RNA enrichment compared to FTA cards and glass microfiber paper^52^. The size of the paper is determined by the volume of solutions and desired analysis time, as discussed previously^53^. After lysis, binding, and washing, RNA is enriched and purified on the paper in the detection unit, which is then taken apart from the mixing unit. The process of using chaotropic agents for RNA extraction/purification is schematically illustrated in Fig. S2 while the detail can be found in the literature^54,55^. Afterward, 25 μL of RT-LAMP amplification mix is loaded into the well of the detection unit, which is then sealed with adhesive tape to prevent evaporation. One way to carry out RT-LAMP is to place it into a commercially available, battery-powered, coffee mug (EmberTM Travel Mug, Ember Technologies, Inc., Westlake Village, CA) (Fig. S3 in *Supplementary Information*) that functions as a water bath at 62.5 °C for isothermal amplification. Another way is to place it into a real-time detector, as discussed in the next section.

The colorimetric detection using SYBR Green was employed at the end of RT-LAMP using naked eye or with the use of a smartphone camera. A blue light-emitting diode (LED) flashlight can be used to enhance the visibility of the color change.

### Real-time detection

Figure 1c shows the real-time detection setup consisting of a digital microscope (AM4117MT-G2FBW, Dino-lite US, Dunwell Tech, Inc) and a portable 3D-printed stand that holds the microscope. The stand has a base that contains a heater, batteries, and an integrated electronic circuit, and it was fabricated using a commercial 3D printer (Ultimaker 3, Geldermalsen, Netherlands) with polylactic acid (PLA) filament. The heater compartment is used to hold the detection unit and ensures that the unit is in contact with the heater, allowing it to maintain a steady temperature of 62.5 °C. The opening in the cover of the heater compartment acts as a pinhole for fluorescence detection while the cover blocks out external light. The microscope is connected to a computer or a smartphone, which collects images every minute. The LED on the microscope is set to turn on for 5 s to take photographs and then turn off for 55 s to avoid possible photobleaching.

The integrated electronic circuit compartment contains the temperature controller, which is used to ensure that the temperature of the detector unit stays at 62.5 °C. The positive temperature coefficient (PTC) heater (Bolsen Tech, USA) is powered by two 9-V batteries and regulated through the temperature controller. The fluorescence intensity of the images taken with the microscope is analyzed using Python code. The average intensity of each image is calculated, and a curve of fluorescence over time is plotted. The threshold time for RT-LAMP is set at 10% above the baseline signal of the amplification curve, and the raw fluorescence amplification plot is smoothed using the moving average plot function in Python^56^.

## Results and discussion

### Effects of primer concentrations on RT-LAMP

We first studied the effects of primer concentrations on RT-LAMP because of the difference between those described in the literature^49^ and what was recommended by the manufacturer, New England Biolabs (NEB). The detail is summarized in Table 1, in which the primer concentrations recommended by NEB are referred to as NEB, their half values (½NEB), and those in the reference (Ref.) paper^49^.

**Table 1:** Primer concentrations tested for RT-LAMP

Primers	NEB (µM)	½NEB (µM)	Ref. paper (µM)
FIP and BIP	16	8	16
F3 and B3	2	1	2
FLP and BLP	4	2	16

**Fig. 2:**
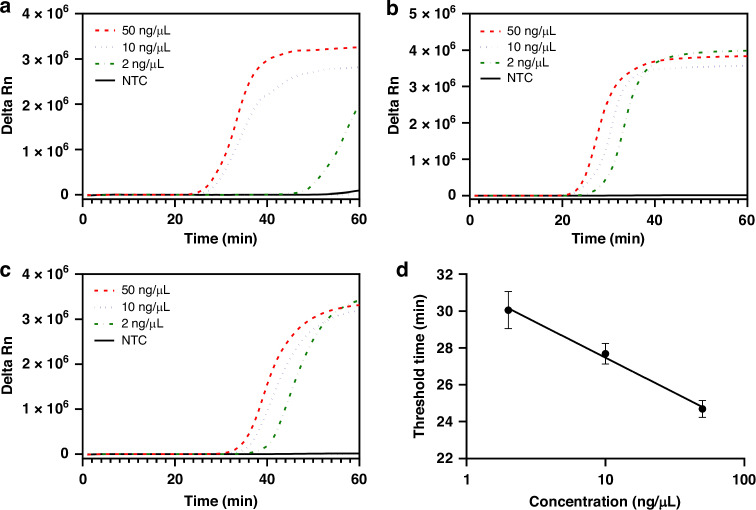
RT-LAMP amplification curves of HIV using different sets of primer concentrations. The changes in fluorescence signals (Delta Rn) are plotted as a function of reaction time while HIV RNA concentrations are 50, 10, and 2 ng/µL, with a no-template control (NTC) included. **a** Primer concentrations at the values recommended by the manufacturer, New England Biolabs (NEB). **b** Primer concentrations at ½NEB. **c** Primer concentrations at the values used in the literature (Hosaka et al.). **d** Calibration curve between the threshold time (Ct) and HIV concentration (in log scale) for the condition in (**b**). The error bars represent one standard deviation of three replicates at each concentration of HIV RNA samples

Figure 2 compares RT-LAMP amplification curves under these three sets of primer concentrations. The set of ½NEB primer concentrations exhibited the shortest amplification time (<35 min) for HIV RNA concentrations of 50, 10, and 2 ng/µL, with no specific amplification observed in NTC for 60 min. In contrast, both the NEB and Ref. paper conditions required >50 min to achieve the signal plateau. The calibration curve between the threshold time (Ct values given by the instrument) and HIV RNA concentration is shown in Fig. 2d, in which ½NEB was used.

The minimum reaction time required to achieve the detection of HIV was then validated for the conditions using ½NEB primer concentration and endpoint detection (i.e., detected only at the end of amplification, without real-time monitoring). 1 µL of each 50, 10, and 2 ng/µL of HIV RNA was added into three 0.2-mL-reaction tubes, followed by adding 24 µL RT-LAMP mix. After incubation of 35 min at 62.5 °C, a dye was added at the end of amplification to carry out the endpoint detection. We observed color changes in all tubes and no color change in the NTC, as shown in Figure S4. Two replicates were carried out to confirm reproducibility. The results indicate that an incubation period of 35 min is sufficient for HIV detection when the endpoint detection is used.

### HIV detection with handheld device

To extract HIV RNA from serum or plasma samples, we utilized 1:4:4 ratio of (sample) to (lysis buffer) to (binding buffer) as recommended by the manufacturer of the RNA preparation kits (Qiagen). For the handheld device in Fig. 1a, we used 140 μL sample and pre-loaded 560 μL of lysis buffer (Buffer AVL, Qiagen), and 560 μL of binding solution (ethanol, Molecular Biology Grade, Fisher BioReagents™), as well as 500 μL each of washing buffer 1 and 2 (Buffer AW1 and AW2, Qiagen) into the respective reservoirs. The process starts with introducing the sample into the mixing unit, followed by sliding it through the buffer unit and releasing the lysis buffer (well 1), binding buffer (well 2), and washing buffers 1 and 2 (wells 3 and 4), in a sequence. The timing of the sequence is controlled by the respective valve in each reservoir. For smaller sample volumes (50 µL) due to the limited sample amount, the 1:4:4 ratio was maintained (i.e., 200 µL of AVL buffer and 200 µL of ethanol). While these liquid solutions pass through the detection unit, RNA is captured and concentrated directly onto the paper pad^50^. This enrichment process is simpler and more efficient than traditional solid-phase extraction (SPE) methods because it eliminates the elution step. Instead, the concentrated and purified RNA on the paper is used directly for amplification.

**Fig. 3:**
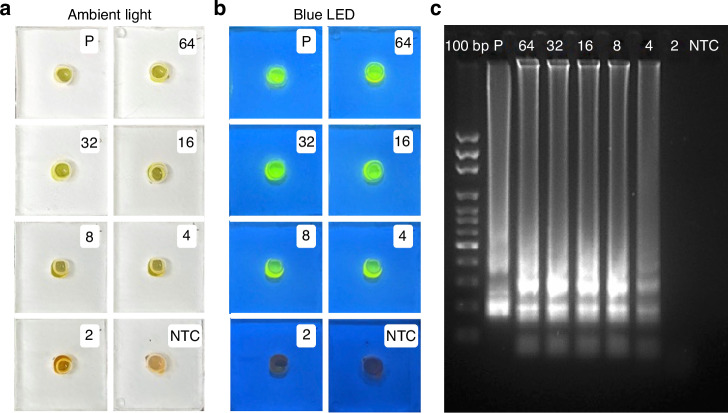
HIV detection using the device in Fig. 1a and serum samples. **a** Pictures of the detection units after 35 min. RT-LAMP using HIV concentrations of 64, 32, 16, 8, 4, and 2 copies of HIV in human serum, under ambient light. P stands for the positive control while NTC is a no-template control. **b** Pictures of the same device in (**a**) under blue LED flashlight. **c** Gel electrophoresis of amplification products in (**a**), where lane 1 is a DNA ladder while other lanes are the same as in (**a**)

We studied the performance of our device by detecting HIV in human serum samples (ARP-3443) with known concentrations of HIV in each 50 μL serum sample. We followed the protocol of loading 50 μL of HIV-containing serum sample, 200 μL of AVL buffer, and 200 μL of ethanol into the device. Afterward, 500 μL of buffer AW1 and 500 μL of buffer AW2 were used to purify RNA, followed by RT-LAMP amplification in the detection units. We ran four replicates for each concentration (64, 32, 16, 8, 4, and 2 copies of HIV in 50 μL serum). These HIV samples were prepared by diluting the stock solution discussed in the *Materials and Methods* and then spiking into HIV-negative human serum. Figure 3 shows the results of colorimetric detection. Our results indicate that only one replicate out of four was positive for 2 copies, while all four replicates were positive for 4 copies and above (Table 2). This demonstrates that the visual limit of detection (LOD) in human serum for our device is 4 copies of HIV.

**Table 2:** Summary of HIV detection in serum samples

Sample	64 copies	32 copies	16 copies	8 copies	4 copies	2 copies	NTC
Serum	4/4	4/4	4/4	4/4	4/4	1/4	0/4

Notes: The results are expressed as (the number of positive results)/(the number of tests). NTC (no-template control) is a negative control

We also compared our LOD with the clinical standard LOD of 20 copies/mL for HIV-1 assay approved for diagnosis by the U.S. Centers for Disease Control and Prevention (CDC) using the sample volume of 140 µL as in most commercial products^57^. We tested concentrations of 50, 40, 30, 25, and 20 copies/mL, with five replicates each, and the results are shown in Table S3 (top row). The results indicate the LOD at 30 copies/mL, which is equivalent to 4.2 copies per reaction, calculated using eq. [1].

1 $${{{Quantity}}}=\left(\frac{30\,{{{copies}}}/{{{mL}}}}{1000\,\mu {{L}}/{{{mL}}}}\right){{\times }}140\,{{\mu }}{{L}}$$

This calculated quantity of 4.2 copies/reaction aligns well with our visual LOD at 4 copies/reaction discussed above.

We further assessed the performance of our device in detecting HIV in plasma samples by spiking HIV particles (400, 40, and 4 copies) into plasma samples. Each sample was processed with our handheld device, which successfully detected 400, 40, and 4 copies of HIV in 35 min while no amplification was observed in the NTC (Figure S6).

### Detection specificity

**Fig. 4:**
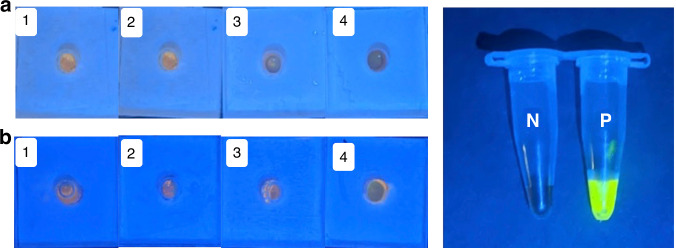
Detection specificity of the platform. Tests of the RT-LAMP detection were carried out using handheld device in Fig. 1a and using HCV sample collected on 09/04/2008 (**a**) and HCV sample collected on 08/31/2004 (**b**). Samples 1-3 are replicates, while sample 4 is a negative control. In addition, the positive control (P) and NTC (N) were carried out in tubes

To assess the specificity of our platform, we conducted experiments using the handheld device to test two deidentifed clinical samples containing hepatitis C virus (HCV). HCV is one of the human RNA viruses that often co-circulate and may interfere with HIV assays^58,59^. Three replicates of each sample were used to perform RT-LAMP in our devices. Among all replicates of the two HCV samples, there was no amplification observed while both the positive control and the NTC behaved as expected, indicating that there was no cross-reactivity of our HIV assay with HCV (Fig. 4). This result is consistent with our similarity check of the HIV primer sequence and viral genomes of both HCV and hepatitis B virus using the basic local alignment search tool (BLAST) from NCBI (Table S2). Our experiments confirmed that our assay is specific for HIV and does not cross-react with HCV (Fig. S5). Given the common occurrence of coinfections of HIV and viral hepatitis, our findings suggest that our assay could be a valuable tool in clinical settings for the detection of HIV in individuals who are also infected with viral hepatitis.

### Validation using clinical samples

To evaluate the handheld device for HIV detection, we tested plasma samples from individuals with suspected HIV and confirmed HCV infections from a prior study. These clinical samples had been previously quantified by PCR-based standard laboratory assay. The samples were provided to researchers in a blinded fashion, labeled only as Sample #1 to Sample #4 without virus amount information. Our handheld device was used to test the samples, and the results were reported back to clinicians who compared them with previous results of the clinical samples.

Each sample vial provided to the researchers contained approximately 200 µL of plasma, which was divided into 50 µL for replicates. In the first round of experiments, HIV RNA was detected in only four out of six test replicates of two positive plasma samples (sample #2 and sample #3), and no false positives were recorded for two negative samples (sample #1 and sample #4), as shown in Fig. S7. During data analysis and comparison of our results with previous PCR tests, we found out that two false negatives were likely related to two experiments that involved device components that did not function properly. In those two devices, we observed leakage from the detection unit, caused by improper adhesion of the double-sided tape to the laminated film holding the paper pad. We have addressed this issue by implementing a thorough inspection of the detection unit before each use.

In the second round, operators were instructed to remove any malfunctioning device and only properly operated experiments should move forward. These sets of deidentified patient samples were labeled as samples #5, #6, #7, and #8. The testing results of this round of clinical samples are shown in Fig. S8 and summarized in Table 3. The results showed that we correctly identified all 3 positive samples and 1 negative sample for HIV. The absence of false-positive and false-negative results in samples #5–8 validated the function of our handheld devices. The results in Table 3 correspond to a positive percent agreement (PPA) of 100.0% (9/9), a negative percent agreement (NPA) of 100% (3/3), and an overall percent agreement (ORA) of 100% (12/12). These results validate the effectiveness of our handheld device for HIV detection at the point of care. Additionally, the device is portable and can be adapted for use in low-resource environments by sealing the buffer unit wells with a PCR tape to prevent spillage during transportation.

**Table 3:** Test result comparison of using the handheld device with PCR

Sample #	Patient #	Date of sample collection	HCV VL (IU/mL)	HIV viral load (copies/mL)	Handheld device
5	Patient 1	9/8/2008	161,880	106,036	3/3
6	Patient 2	7/22/2008	611,221	26,487	3/3
7	Patient 3	1/24/2008	1,242,991	58,224	3/3
8	Patient 4	9/10/2003	3,921,756	< 75^a^	0/3

^a^undetectable HIV, which was labeled as “< 75”. HCV and HIV viral load (VL) were measured by commercial labs

### Integration with a real-time detector

While colorimetric detection described above is convenient for visual assessment, it must be carried out at the end of the incubation period (i.e., endpoint detection). A real-time detector can tell a positive sample as soon as the signal is statistically significantly higher than the background, thus making the detection much sooner than the endpoint detection. In addition, the threshold amplification time can be used to quantify the viral load. Real-time LAMP has been explored for quantitative detection by Notomi and his colleagues in 2004^60^ and by many others^43,61^ thereafter. As a result, we developed a real-time detector and integrated it with our device for HIV detection.

As illustrated in Fig. 1c, the real-time detector consists of a handheld, flashlight-shaped digital microscope and a portable 3D-printed stand. The stand contains a base with three chambers for a heater, batteries, and an integrated electronic circuit (IEC). The heater chamber is for holding the detection unit and it is aligned with the microscope. The IEC chamber is for the temperature controller, which regulates the heater temperature that is powered by batteries. The detector is highly portable, with a total weight less than 1 kg. Importantly, it operates on two 9-V batteries, eliminating the need for an electricity outlet.

We first validated the accuracy and effectiveness of the heater by embedding a T-type thermocouple in the detection unit to measure the temperature after the real-time detector was connected. Figure S9 shows the recorded temperature profile, indicating that the temperature reached the optimal 62.5 °C within 5 min and remained within the optimal RT-LAMP temperature (55–70 °C)^62^. In our experiments, the heater was turned on 5 min before its use, maintaining temperature equilibrium for accuracy and reliability.

**Fig. 5:**
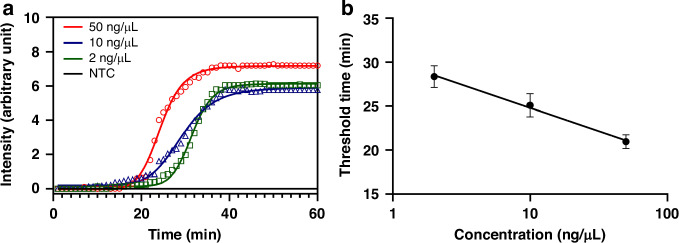
Demonstration of the portable real-time detector for HIV testing. **a** Amplification curves of HIV using the detector in Fig. 1c. Various HIV concentrations were used while NTC is a negative control. **b** Calibration curve between the threshold time (Ct) of RT-LAMP and the HIV concentration (log scale). The error bars are one standard deviation of three replicates at each HIV concentration

To assess the performance of our real-time detector, we performed experiments using HIV RNA samples at varying concentrations, including 50, 10, and 2 ng/µL. As discussed above, the samples were prepared using our handheld device in Fig. 1a. The resulting detection unit containing enriched RNA was placed in the heater compartment of the real-time detector in Fig. 1c. Real-time amplification was monitored and images were processed using Python to create amplification curves, as shown in Fig. 5a. The amplification signal could be observed at 20 min. for HIV at 50 ng/µL, without a need to wait until 35 min. as we did using the endpoint detection. The relationship between the threshold time of RT-LAMP and HIV concentration is shown in Fig. 5b. The results indicate that the handheld system with sample preparation and real-time detection is capable of providing quantitative information on the HIV viral load.

Importantly, the results obtained from our real-time detector (Fig. 5) were comparable to those obtained using a commercial PCR machine (Fig. 2). The slope of the calibration curve in Fig. 5b is quite similar to the one in Fig. 2d. These findings provide strong evidence of the potential of our integrated system to accurately quantify the viral load of HIV RNA samples.

Our device is cost-effective for commercialization, with a total cost of $5.41 per test using endpoint detection, as shown in Table S4. This cost includes $1.25 for the 3D-printed handheld device and $4.16 for reagents and RT-LAMP mixture. These costs are based on retail prices we paid, and they will be likely lower for manufacturing when they are bought at the wholesale price. For real-time detection, an additional one-time investment of $1003 for the real-time detector is required. Alternative detectors at lower price are available, but this cost overweighs the benefits of detection sensitivity.

## Conclusion

We have successfully developed a handheld HIV detection platform, which integrates valve-enabled sample preparation and RT-LAMP isothermal amplification with a real-time detector. Our platform demonstrated comparable virus preparation to commercial RNA extraction kits but with the added advantage of not requiring lab equipment such as centrifuges and pipettes, making it an ideal choice for POC use. The integration of our handheld sample preparation device with a real-time detector enables sample-to-answer HIV virus detection and viral load quantification at the POC, demonstrating its potential to be used in resource-limited settings.

We evaluated our device using both human serum samples with known HIV concentration and human plasma samples with known HCV. Our device showed 100% specificity and no cross-reactivity with HCV, making it ideal for HIV diagnosis. Our device had a detection limit of 30 copies/mL for HIV, indicating its true usefulness considering that the peak viral loads during acute HIV infection are often greater than 100,000 copies/mL^22,63,64^. This detection limit is comparable to that of commercial instruments used in clinical settings. In contrast to the standard RT-PCR analysis, which typically takes 2–3 h, our device employs RT-LAMP isothermal amplification, enabling the detection of HIV in clinical samples in as short as 60 min (40 min of sample preparation and 20 min of real-time detection, or 75 min if using 35 min. endpoint detection). The reagent used in our handheld sample preparation unit is commercially available and can be stored at room temperature. Currently, the RT-LAMP mix is preloaded in disposable pipettes and stored in an ice box. In the future, we aim to integrate lyophilized RT-LAMP reagents into the device, eliminating the need for cold storage. It should be noted that there are other RT-LAMP-based HIV detection devices, though there are differences between them and this work as discussed in the *Introduction*.

Our device has the potential to improve HIV detection, especially in acutely infected individuals who may be missed by antibody-based strip tests. The speed, sensitivity, and specificity of our platform make it an attractive alternative in certain field and resource-limited settings. In addition, this device can be adapted for detection of other pathogens including mosquito-borne pathogens such as Zika and Dengue viruses.

## Supplementary information

Supplementary Information - Marked Up copy
