# Supplementary material for: A handheld HIV detection platform using paper-based sample preparation and real-time isothermal amplification
Source: Microsyst Nanoeng. 2024 Nov 29;10:181. doi: 10.1038/s41378-024-00822-1 (PMC11607462; doi:10.1038/s41378-024-00822-1)
Supplement: Supplementary file 1 — Supplementary Information - Marked Up copy [file 41378_2024_822_MOESM1_ESM.pdf]

Supplementary Information

A Handheld HIV Detection Platform using Paper-Based Sample Preparation and Real-Time Isothermal Amplification

George Adedokun[1], Gurjit Sidhu[2], Morteza Alipanah[1], Gary P. Wang[2,3,*], and Z. Hugh Fan[1,4,*]

[1]Interdisciplinary Microsystems Group, Department of Mechanical & Aerospace Engineering, University of Florida, Gainesville, FL 32611, USA

[2]Division of Infectious Diseases and Global Medicine, College of Medicine, University of Florida, Gainesville, Florida 32610, USA.

[3]Medical Service, North Florida/South Georgia Veterans Health System, Gainesville, Florida 32608

[4]Purrit Family Department of Biomedical Engineering, University of Florida, Gainesville, FL 32611, USA

*Authors to whom correspondence should be addressed. e-mail: hfan@ufl.edu; gary.wang@medicine.ufl.edu.

Table S1. Sequences of RT-LAMP primers used for HIV detection*

Primer name	Sequence (5' - 3')	Genome position
**F3**	GGTAAGAGATCAGGCTGAACATC	4721–4743
**F2**	AGACAGCAGTACAAATGGCA	4747–4766
**FLP**	TTAAAATTGTGGATGAAT	4786–4769
**F1c**	CCCCAATCCCCCCTTTTCTT	4806–4787
**B1c**	AGTGCAGGGGAAAGAATAGTAGAC	4812–4835
**BLP**	GCAACAGACATACAAACTAAAG	4842–4863
**B2**	CTGCTGTCCCTGTAATAAACCC	4921–4900
**B3**	GCTGGTCCTTTCCAAAGTGG	4945–4926
**FIP**	F1c + F2	
**BIP**	B1c + B2	

*Hosaka, N. *et al.* Rapid detection of human immunodeficiency virus type 1 group M by a reverse transcription-loop-mediated isothermal amplification assay. *Journal of Virological Methods* **157**, 195–199 (2009).

```
4681 tagaatctat gaataaagaa ttaaagaaaa ttataggaca ggtaagagat caggctgaac
                                                              F3
4741 atcttaagac agcagtacaa atggcagtat tcatccacaa ttttaaaaga aaagggggga
                  F2                    LF                    F1
4801 ttggggggta cagtgcaggg gaaagaatag tagacataat agcaacagac atacaaacta
                       B1c                                    LB
4861 aagaattaca aaaacaaatt acaaaaattc aaaattttcg ggtttattac agggacagca
                                                              B2
4921 gaaatccact ttggaaagga ccagcaaagc tcctctggaa aggtgaaggg gcagtagtaa
                  B3
```

Sequences and location of RT-LAMP primers related to "Human immunodeficiency virus type 1 (HXB2), complete genome; HIV1/HTLV-III/LAV reference genome" – GenBank: K03455.1 targeting pol-integrase gene, a well-conserved region of HIV-1genome.

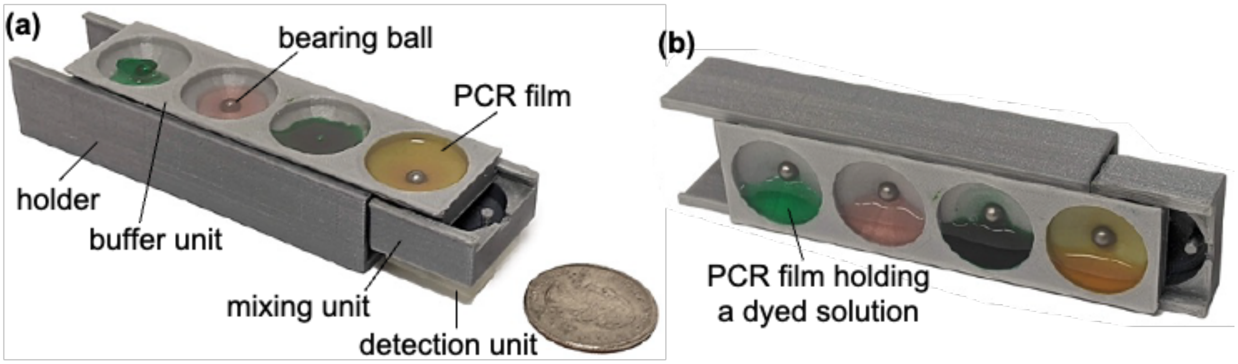

Figure S1. Photographs of an assembled device with a holder that provides support for the device. (a) the device was sealed with a PCR tape to prevent spill during transportation though the transparent PCR film is difficult to observe in the photograph; (b) the same device of (a) is rotated to show dye solutions are held by the PCR film. The total time for using the device to process a plasma sample of 140 µL is approximately 40 minutes: the lysis and binding steps about 15 to 18 minutes and washing buffer 1 and 2 about 12 minutes each. The total time reduced to about 25 minutes when 50 µL plasma samples were used. Note that samples with viscosity less than plasma (e.g., urine) will flow through the device even faster. As to reusability, the 3D-printed buffer and mixing units can be used again after they are thoroughly cleaned and sterilized.

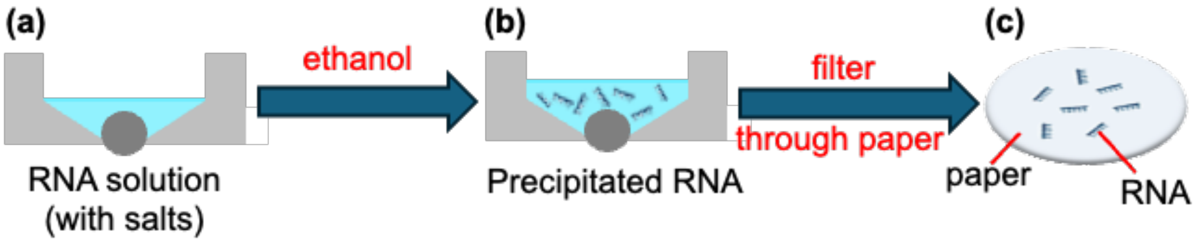

Figure S2. Schematic illustration of RNA enrichment by ethanol. (a) After lysis, RNA molecules are dissolved in the solution containing salts. Negatively charged phosphate groups in the RNA backbone are highly hydrophilic, attracting water molecules to form a hydration shell. (b) After ethanol is added as a chaotropic agent, it disrupts the hydration shell, allowing the negative charges on unshielded phosphates to be neutralized by positive charges on cations in the solution. As a result, RNA molecules become hydrophobic, causing them to precipitate. (c) When the solution is filtered through a chromatography paper, RNA precipitates are retained while the rest of solution is removed. After washing, RNA is purified and ready for the amplification step.

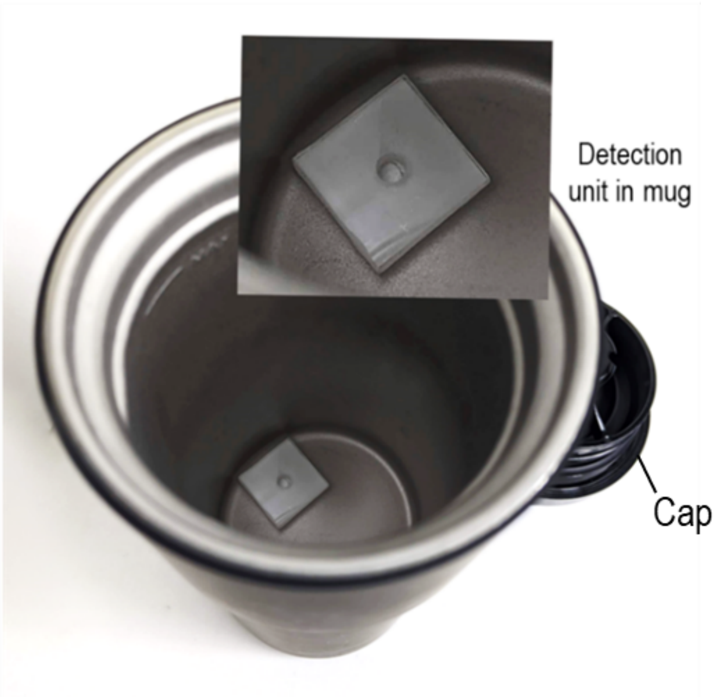

Figure S3. Detection unit in the Ember travel mug for amplification at 62.5 °C. The mug is capped during incubation. A display shows the temperature. A close-up picture of the detection unit is shown at the top.

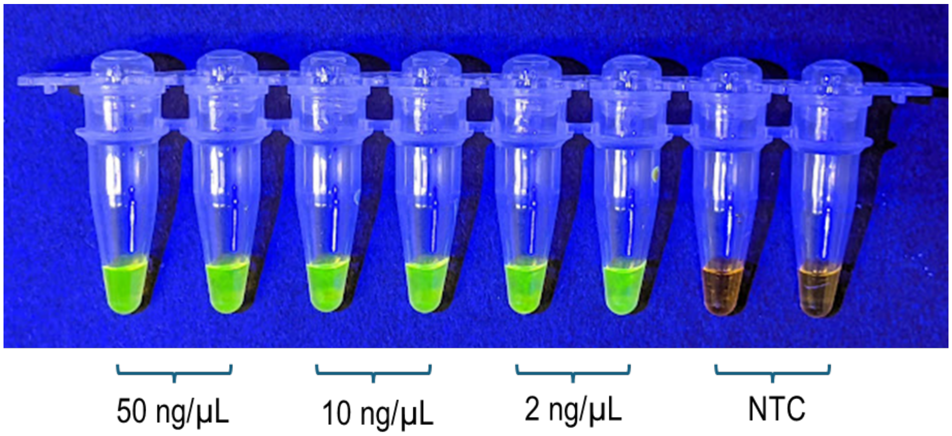

Figure S4. RT-LAMP endpoint detection of 50, 10, and 2 ng/μL of HIV RNA. The endpoint detection was carried out after 35 min of RT-LAMP reactions. NTC stands for no-template control.

Table S2: HIV primer sequences' similarity analysis with HCV and HBV genome using BLAST

Primer	HCV	HBV
F3	None	None
B3	None	None
FIP	None	None
BIP	None	None
FLP	None	None
BLP	None	None

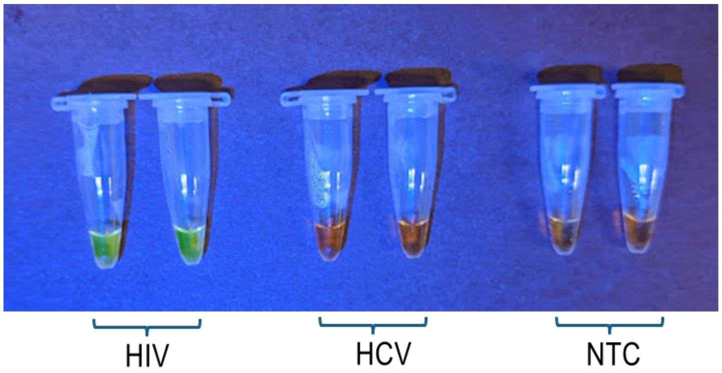

Figure S5. Specificity test of using RT-LAMP detection assay of HIV against HCV RNA. A no-template control (NTC) was used simultaneously.

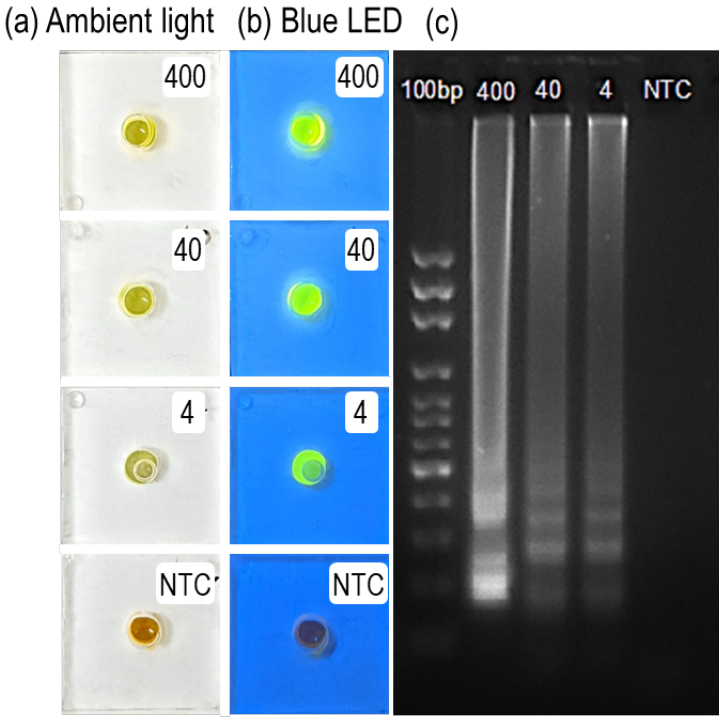

Figure S6. (**a**) Pictures of the detection units containing 400, 40, and 4 copies of HIV spiked in human plasma samples, photographed using a smartphone under ambient light. NTC is no-template control. (**b**) Picture of the same device in (a) under a blue LED flashlight. (**c**) Gel electrophoresis of the amplification products from the RT-LAMP reactions in (a). Lane 1 is a DNA ladder while other lanes are the same as in (a).

Table S3. Summary of HIV detection in serum and plasma samples.

**Sample**	**50 cp/mL**	**40 cp/mL**	**30 cp/mL**	**25 cp/mL**	**20 cp/mL**	**NTC**
Serum	5/5	5/5	5/5	0/5	1/5	0/5
			**400 copies**	**40 copies**	**4 copies**	**NTC**
Plasma			2/2	6/6	5/6	0/6

Note: The results are shown as (the number of positive results)/(the number of tests). NTC (no-template control) is a negative control. cp/mL: copies/mL. Note that 140 μL of sample volume was used for detecting HIV in serum samples whereas 50 μL of plasma samples were used which contained 400, 40, or 4 copies of HIV particles.

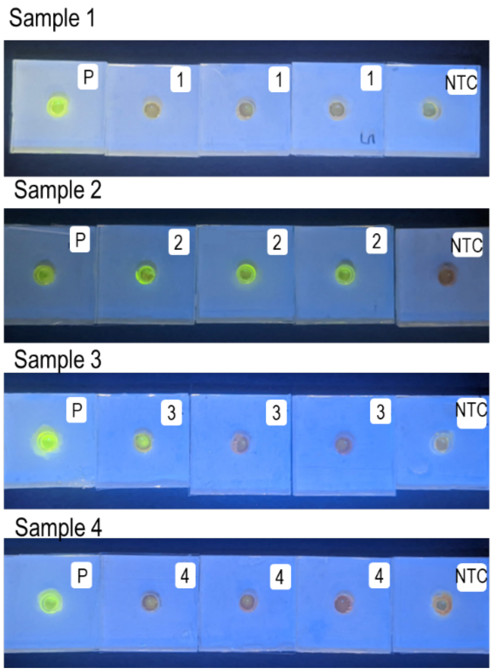

Figure S7. Picture of detection unit for HIV detection in de-identified clinical samples #1-4. For each sample test, there is a positive control (P), 3 sample replicates, and a no-template-control (NTC).

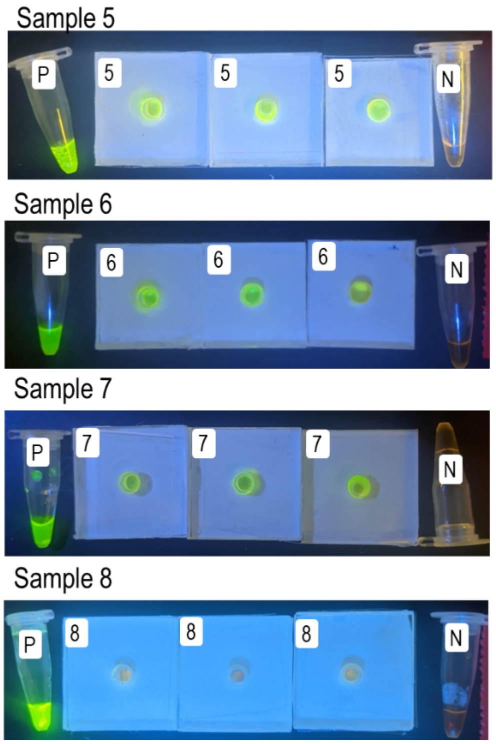

Figure S8. Pictures of detection units for HIV detection in de-identified clinical samples #5-8. For each sample test, there is a positive control (P), 3 sample replicates, and a no-template-control (NTC).

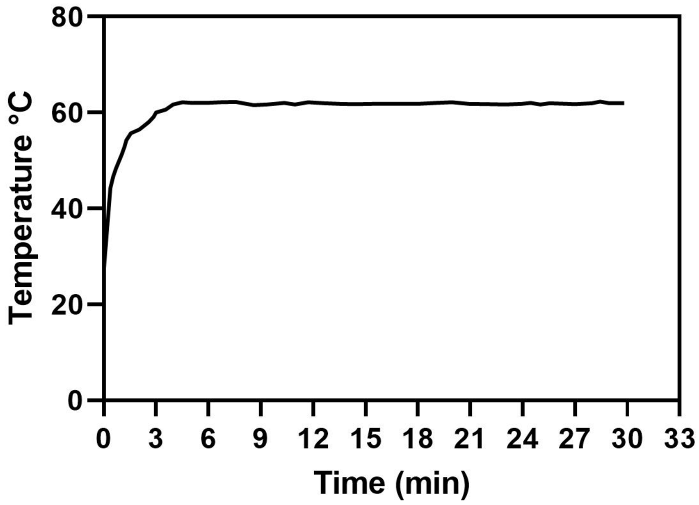

Figure S9. Temperature profile of a T-type thermocouple that was placed inside the detection unit. The heater was turned on after the detection unit was placed onto the real-time detector in Figure 1c.

Table S4: Cost analysis per device and assay for the handheld device

Material	Cost (bulk quantity)	Quantity/device	Cost/device ($)
PLA	$23/1 kg	30	0.69
Polycarbonate sheet	$12/sheet (~200 units/sheet)	1	0.06
Chromatography paper	$0.61/sheet (~1,600 paper pads/sheet)	1	0.01
Thermoplastic films	$33/roll (~2,300 laminations/roll)	2	0.03
Balls (valves)	$5.54/50 balls	4	0.45
		**Total cost (Device)**	**1.25**
		**Quantity/reaction**	**Cost/reaction ($)**
dNTPs	$70/500 µL	3.5 µL	0.49
Isothermal buffer	$21/6 mL	2.5 µL	0.01
HIV primers	$253.37/~1.2 mL	2.5 µL	0.53
MgSO4	$23/6 mL	1.5 µL	0.01
Bst 2.0	$140/200 µL	1 µL	0.7
WarmStart RTx	$235.45/125 µL	0.5 µL	0.94
UDG	$268.6/500 µL	0.5 µL	0.27
dUTP	$52.70/700 µL	0.5 µL	0.04
SYBR Green	$350/500 µL	0.5 µL	0.35
Nuclease free water	$35/100 mL	13 µL	0.01
AVL lysis buffer	$126.75/155 mL	0.56 mL	0.46
Ethanol	$33/500 mL	0.56 mL	0.04
AW1	$86/242 mL	0.5 mL	0.18
AW2	$86/324 mL	0.5 mL	0.13
		**Total/reaction**	**$4.16**
		**GRAND TOTAL**	**$5.41**
